# Advanced Manual Therapy Approach for the Management of Non-specific Low Back Pain: A Case Report

**DOI:** 10.7759/cureus.28727

**Published:** 2022-09-03

**Authors:** Neha Chitale, Deepali S Patil, Pratik Phansopkar

**Affiliations:** 1 Department of Musculoskeletal Physiotherapy, Ravi Nair Physiotherapy College, Datta Meghe Institute of Medical Sciences, Wardha, IND; 2 Department of Kinesiotherapy & Physical Diagnosis, Ravi Nair Physiotherapy College, Datta Meghe Institute of Medical Sciences, Wardha, IND

**Keywords:** rehabilitation, integrated neuromuscular inhibition, physical therapy rehabilitation, chronic non-specific low-back pain, chronic low back pain (clbp)

## Abstract

The lower back is a complex area, with joints having a very limited range of motion and vast muscular activity. Low back pain is common, and its management depends upon the pain generators. There are various treatment options available, depending on the cause of the pain. In this case, a medical student with pain on a scale of 8/10 on activity came to the physiotherapy department for rehabilitation and pain relief. Her low back pain was chronic and non-specific. The aim of rehabilitation was to reduce the pain and make the patient pain-free. An integrated neuromuscular inhibition technique (INIT), along with a conventional physiotherapy approach, was given to the patient. The integrated neuromuscular inhibition technique can be used to reduce lower back pain and functional disability. After physiotherapy rehabilitation, the range of lumbar flexion improved. In patients presenting with low back pain, early physiotherapy should be started to maintain strength, reduce pain, and reduce functional disability.

## Introduction

The lower back region consists of the lumbar and sacral regions of the spine. Low back pain (LBP) is one of the most common conditions, with about 50 percent of the population encountering pain in the back region at least once a year [1]. Low back pain affects activities that are necessary for everyday living and makes them significantly more difficult to perform. Pain can be at the lumbar, lumbosacral, or sacroiliac level, depending upon the region involved [2]. Depending on the cause, low back pain can be classified as specific or non-specific [3]. Low back pain can be categorized as being acute (0-14 days), sub-acute (two-12 weeks), or chronic (>three months) depending on the duration [3-5]. LBP should be managed at an earlier stage as it will lead to more biomechanical alterations in the future. Various non-pharmacological modalities should be used, and the best method to manage low back pain should be utilized. The integrated neuromuscular inhibition technique (INIT) is one of the techniques that can be used to manage LBP. The INIT method involves three distinct manual maneuvers [6]. The three techniques are trigger point release, strain counterstrain technique, and muscle energy technique (MET). In a trigger point release, compression is applied to the trigger point region and held for 15 seconds, whereas in the strain counterstrain technique, the superficial fascia is stretched. MET works on the principle of reciprocal inhibition. As it uses three distinct manual techniques, it may have a significant effect on reducing low back pain. In this case report, INIT was used as a treatment approach to reduce LBP.

## Case presentation

A 22-year-old female, a medical student by profession, came to the physiotherapy department with the complaint of LBP. The patient was suffering from low back pain for the last 4 months, which was less in the morning and increased with prolonged standing or any activity. The patient had a history of heavy lifting, and her profession involved prolonged sitting. The onset of pain was sudden, with a gradual progression. Because of LBP, the patient was not able to sit for a long time and was having difficulty performing daily activities. The patient went to an orthopedic surgeon for the same reason. An MRI was recommended and a spasm in the paraspinal region with thoracolumbar fascia tightness was identified. Pain medications were given, but the pain didn’t subside, hence she was referred to the physiotherapy department.

Clinical findings

The patient was examined in a prone position. Grade 3 tenderness was present laterally in the lumbar spine in the paraspinal region. A spasm was present in paraspinal muscles, and trigger points were present in the quadratus lumborum. She had a dull pain in the region. On the numeric pain rating scale, the pain was 5/10 during rest and 8/10 at the time of activity. On the modified Oswestry disability index, the patient had a 70% disability because of low back pain. The modified Schober test showed 4.6 cm of lumbar flexion. The MRI, which was done on February 9, 2021, showed normal vertebral height and disc height. The alignment of the bone was normal. The slump test was negative.

Management

**Figure 1:**
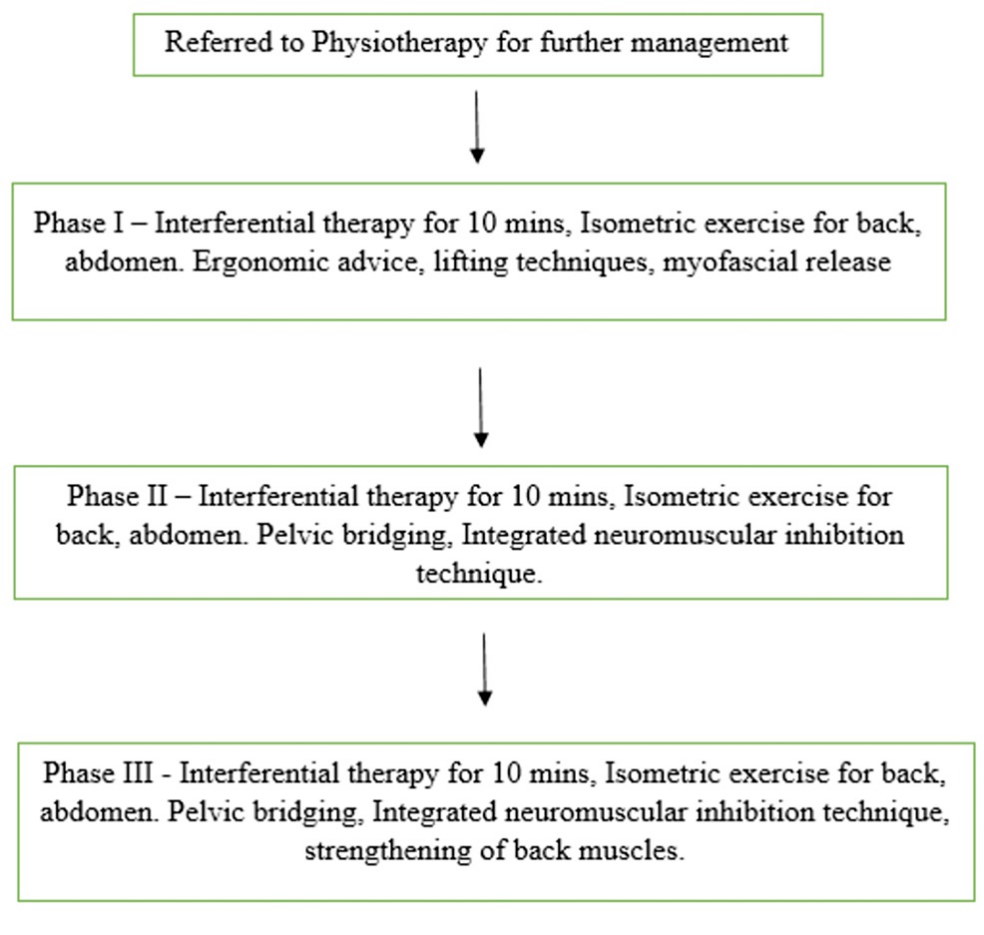
A flowchart showing the rehabilitation protocol for the patient

Physiotherapy rehabilitation was done for six weeks with five sessions per week. Figure 1 shows a flowchart with the rehabilitation protocol for the patient. 

**Figure 2:**
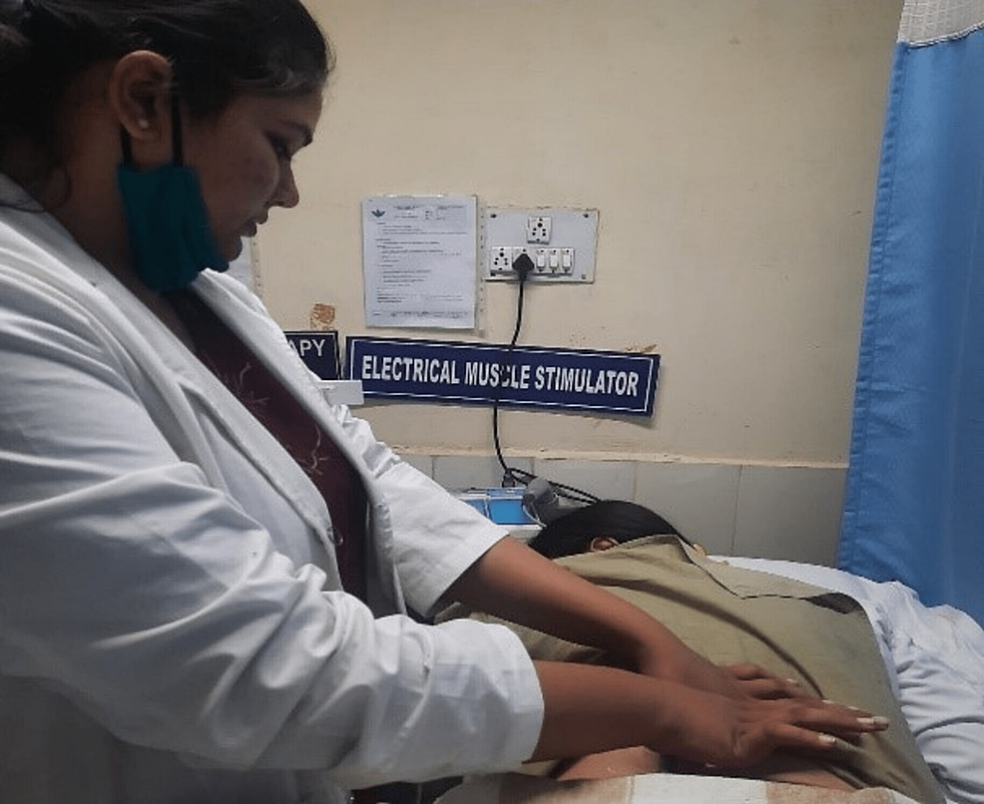
Ischemic compression for trigger point release Trigger points were identified in the quadratus lumborum region, and sustained direct pressure with the thumb was applied till the pain was modified.

Phase 1 (week one to week 2) focused on patient education, ergonomic advice, pain reduction, and maintaining the strength of back muscles. The patient was educated on the condition, and the dos and don’ts were explained. Proper lifting technique was taught to the patient. For pain management, interferential therapy (IFT) was given for 10 minutes on four pole in a sweep pattern. Isometric exercises for the back and abdomen were taught, and the patient was asked to repeat them 10 times with a hold of 10 seconds two times a day. Trigger points were identified, and trigger point release was done via ischemic compression (Figure 2). The strain counterstrain method was used post-trigger point release.

Phase 2 (week two to week four) concentrated on the gains made in Phase 1 as well as the strengthening of the back muscles. The same rehabilitation protocol was continued as in Phase 1. In Phase 2, the integrated neuromuscular inhibition technique (INIT) was used, and the muscle energy technique (MET) was used for the quadratus lumborum. The reciprocal inhibition technique (RIT), a type of MET, was used for three repetitions. Myofascial release in the form of trigger point release was continued along with the strain counterstrain technique.

Phase 3 (week four to week six) was focused on maintaining and strengthening the functions and strengths established in Phase 2. In Phase 3, strengthening exercises for back muscles were prescribed along with core-strengthening exercises. As a part of strengthening exercises, the pelvic bridge exercise was prescribed to the patient, and she was asked to do 10 repetitions with a 10-second hold. This was followed by the unilateral pelvic bridge exercise performed for 10 repetitions on each side with a 10-second hold. Curl-ups were taught to the patient, and she was asked to perform 10 repetitions of the exercise.

A summary of the phase-wise treatment plan along with goals and therapeutic interventions is shown in Table 1.

**Table 1:** A summary of the phase-wise treatment plan along with goals and therapeutic interventions

Phase	Goal	Therapeutic intervention
Phase 1 (week one to week two)	Patient education, ergonomic advice, and pain reduction	Proper lifting method taught along with dos and don’ts, interferential therapy for 10 minutes on four pole in a sweep pattern, trigger point release using ischemic compression.
Phase 2 (week two to week four)	Strengthening of the back muscles	Muscle energy technique was used for three repetitions, strain counterstrain technique and ischemic compression for trigger point release, and isometric exercises were taught for strengthening the back and the abdomen.
Phase 3 (week four to week six)	Maintaining achieved strength and increasing strength	Pelvic Bridges, unilateral pelvic bridges, and curl-ups were taught to strengthen the back and core.

After six weeks, a home exercise program including core-strengthening exercises was given. In physiotherapy management, the main aim was to reduce pain, decrease functional disability, and improve the lumbar range of motion. 

Follow-up and outcome measures

After the completion of the physiotherapy treatment, a two-month follow-up was performed. A home protocol was designed for the patient, and changes in the home protocol were made according to the assessment done at the time of follow-up. The modified Oswestry disability index taken before the physiotherapy treatment showed 70% disability, while post-treatment, 30% disability was seen. A modified Schober test showed 4.6 cm of lumbar flexion before physiotherapy sessions. Post-physiotherapy, 6.1 cm of lumbar flexion was seen.

## Discussion

This case report is of a 22-year-old female with low back pain that was nonspecific and chronic. The goal of rehabilitation was to reduce pain and make the patient pain-free [7]. The cause of the pain was muscular as there were triggers present in the quadratus lumborum that were restricting the range of motion and thus leading to pain. In a systematic review, it was concluded that an increase in flexibility in the back will lead to an increase in the range of motion [8]. Flexibility was increased with the help of muscle energy techniques and strain counterstrain techniques, whereas trigger points were treated with ischemic compression.

For long-term effects, core-strengthening exercises should be given to maintain strength. Chronic low back pain may reoccur after treatment is discontinued, so core stability is important in this regard. Evidence suggests that core strengthening has a significant effect on pain levels in patients with LBP [9]. The strengthening of the back and lower limb muscles, when compared with core muscle strengthening, was less effective in treating pain [10]. Non-specific low back pain is a vast term, and it contains various aspects in which core strengthening plays a major role [11].

In a 2020 randomized controlled trial, Santosh C. Metgud et al investigated the effect of INIT on trigger points in patients with LBP. The study included 44 patients with LBP. Stretching and strengthening were prescribed for Group A, while INIT was used for Group B. With regard to conventional physiotherapy methods, both groups received therapeutic ultrasound and hot fomentation. This treatment was done consecutively for five days. Pain pressure, functional disability, and range of motion were the outcomes assessed before and at the end of the fifth session. The authors came to the conclusion that INIT has a better effect on reducing myofascial trigger point tenderness and thus low back pain [12]. INIT is a combination of three techniques; therefore, it gives a combined effect of all three techniques and hence reduces low back pain.

## Conclusions

Physiotherapy has a significant effect on maintaining and increasing strength. The integrated neuromuscular inhibition technique can be used to reduce low back pain and functional disability. Physiotherapy should be started early to maintain strength and reduce pain. INIT is a combination of MET, strain counterstrain, and ischemic compression. Ischemic compression helped treat myofascial trigger points, MET helped with muscle strength and tightness, and the strain counterstrain technique helped with both myofascial and muscle tightness. The combined effect of all three techniques helped reduce low back pain.

While pain and functional disability were reduced, an increase in the lumbar range of motion has also been noted post-rehabilitation. Apart from pain and muscle tightness, there are many factors that can affect the range of motion. With INIT, both pain and muscle tightness were addressed, and the range of motion significantly improved.
